# MicroRNAs Contribute to Breast Cancer Invasiveness

**DOI:** 10.3390/cells8111361

**Published:** 2019-10-31

**Authors:** Ivana Fridrichova, Iveta Zmetakova

**Affiliations:** Cancer Research Institute, Biomedical Research Center, Slovak Academy of Sciences, 845 05 Bratislava, Slovakia; Iveta.Zmetakova@savba.sk

**Keywords:** miRNA expression, miRNA target genes, epigenetic regulation, female cancer, breast cancer, invasiveness, metastasis

## Abstract

Cancer statistics in 2018 highlight an 8.6 million incidence in female cancers, and 4.2 million cancer deaths globally. Moreover, breast cancer is the most frequent malignancy in females and twenty percent of these develop metastasis. This provides only a small chance for successful therapy, and identification of new molecular markers for the diagnosis and prognostic prediction of metastatic disease and development of innovative therapeutic molecules are therefore urgently required. Differentially expressed microRNAs (miRNAs) in cancers cause multiple changes in the expression of the tumorigenesis-promoting genes which have mostly been investigated in breast cancers. Herein, we summarize recent data on breast cancer-specific miRNA expression profiles and their participation in regulating invasive processes, in association with changes in cytoskeletal structure, cell-cell adhesion junctions, cancer cell-extracellular matrix interactions, tumor microenvironments, epithelial-to-mesenchymal transitions and cancer cell stem abilities. We then focused on the epigenetic regulation of individual miRNAs and their modified interactions with other regulatory genes, and reviewed the function of miRNA isoforms and exosome-mediated miRNA transfer in cancer invasiveness. Although research into miRNA’s function in cancer is still ongoing, results herein contribute to improved metastatic cancer management.

## 1. Introduction

GLOBOCAN 2018 statistical estimates from the International Agency for Research on Cancer reveal almost 8.6 million new female cancer cases and 4.2 million associated deaths. Therein, cancers of the breast, cervix uteri, mostly endometrial corpus uteri, ovary, vulva, and vagina rated 24.2%, 6.6%, 4.4%, 3.4%, 0.5% and 0.2% incidence rates and 15%, 7.4%, 2.1%, 4.4%, 0.4% and 0.2% mortality rates, respectively [1]. Comparison of the statistics and spread of these tumors show the following.

While almost one-fifth of breast cancer patients (BC) develop metastatic disease, at initial diagnosis approximately 6% had distant metastases in the bone, liver, lung, and non-axillary lymph nodes and less in the brain [2,3,4]. In contrast, thirteen percent of cervical cancer sufferers (CC) were classified as advanced, and their metastases through the haematogenous and lymphogenous systems have different treatment and survival rates. CC can spread into the peritoneal lymph nodes and distant organs, such as the lung, liver, bone, and brain [5]. Ovarian cancer (OC) has the second highest mortality rate of all gynaecological malignancies and approximately 75% of patients have cancer cell dissemination in the peritoneal cavity at the time of diagnosis [6]. These tumors were mostly epithelial and usually primary, but 10–20% were diagnosed as ovarian metastases of breast, colorectal, endometrial, stomach, or appendix cancer [7]. Finally, endometrial cancer (EC) is frequently diagnosed at an early stage when the malignant changes are still confined to the uterus, and the five-year overall survival in patients without metastasis ranges from 74% to 91%, because of high prevalence of less aggressive endometrioid type [8]. Non-endometrioid malignancies tend to have early metastatic spread, into the cervix, vagina and myometrium, and distant metastases are commonly found in the lungs. Similar to OCs, rare EC’s have been identified as endometrial metastases originating from breast, ovarian, lung, stomach, and colorectal cancers and melanomas [9]. Metastatic disease becomes a very serious medical problem because metastases are usually resistant to conventional therapy, and only palliative therapeutic options remain available.

Metastasis development is also known as the invasion-metastasis cascade and it involves a complex process defined in distinct steps. The cancer cells intravasate locally through surrounding extracellular matrix and stromal cell layers and enter the lumina of adjacent lymph and blood vessels, where they survive and are transported through the circulatory system evading physical damage and host immune response. The cancer cells are then arrested or adhere to vessel walls and extravasate into the parenchyma of distant organ, where surviving cancer cells form micro-metastases and proliferate into macro-metastases to form a metastatic colonization [10,11]. During these processes, cancer cells and non-malignant cells in the tumor microenvironment (TM) undergo genetic and epigenetic changes [12].

Metastasis appears highly inefficient because less than 0.01% of cancer cells in the systemic circulation ultimately develop into macroscopic metastases [13]. Increasing knowledge of cancer and non-malignant cells’ molecular characteristics and interactions supports early dissemination of semi-competent metastatic cancer cells from a primary tumor, accumulating diverse molecular changes at distant body sites rather than late dissemination of fully malignant cells [12,14,15]. These disseminated cancer cells can also remain dormant for months and years before causing later cancer recurrence [16]. Although initiation of proliferating dormant cancer cells remains unclear, it may be regulated by the interaction of cancer cells with microenvironmental components, blood supply limitations, or an active immune system [10].

For patients with early cancer lesions, an understanding of the mechanisms allowing the physical relocation of cancer cells from primary tumors is likely to be helpful for preventing metastasis, and for patients with metastatic seeding greater knowledge of the mechanisms leading to successful colonization of cancer cells should contribute to the development of more effective therapy [17].

## 2. MicroRNA Biogenesis

A new mechanism mediated by approximately 22 nucleotide-long non-coding RNA molecules known as microRNAs (miRNAs) which underlies epigenetic regulation of gene expression was discovered two decades ago. Most miRNAs are highly conserved in different mammalian species and are capable of regulating key biological processes, including differentiation, development and cell proliferation, in a sequence-specific manner [18]. The latest 9/2017 miRTarbase update confirms 2599 human miRNA molecules with 15,064 target genes (http://mirtarbase.mbc.nctu.edu.tw/php/statistics.php). Of 380,639 miRNA-target interactions, 5831, 7676, 12,886 and 359,298 have been experimentally validated by Western blot, luciferase assays, microarrays, and next-generation sequencing, respectively [19].

While the 6/2016 updated version 2.0 of the Database of Differentially Expressed miRNAs in Human Cancers documented that the highest number of up- and down-regulated miRNAs in all female malignancies were identified in BC than in any gynaecological cancer (http://www.picb.ac.cn/dbDEMC/statistics.html) [20], the more detailed 02/2009 updated cancer-specific miRNA database identified 507, 188, 66, and 202 instances of changed miRNA expression in BC, CC, EC and OC (http://mircancer.ecu.edu) [21]. With this research established, we focused herein on the current knowledge of aberrant miRNA expression profiles associated with specific invasive processes in breast malignant transformation. The miRNA sequences are frequently located in introns and very rarely found in exons, and these can share a promoter with the host gene or they can have an independent promoter and be independently transcriptionally regulated when situated in inter-genic regions. Further, the miRNAs are organised as either single units or tandemly in bi- or poly-cistronic clusters and they are present in all human chromosomes except Y [22]. Most are transcribed by RNA polymerase II in the nucleus as several-hundred-nucleotide-long primary miRNA transcripts (pri-miRNAs) containing a 5′ prime cap and a 3′ poly(A) tail [23]. This precursor is cleaved during micro-processing by drosha ribonuclease III (DROSHA) and its cofactor, the double-stranded-RNA-binding-protein DiGeorge syndrome critical region 8 (DGCR8) microprocessor complex subunit. This results in 60–70 nucleotide-long precursor miRNAs (pre-miRNAs) with hairpin loop structures [24]. The pre-miRNAs are then actively exported from the nucleus to the cytoplasm by exportin-5 (XPO5)/Ran guanosine triphosphate (GTP) transporter [25,26], and the pre-miRNA is subsequently cleaved near the terminal loop by ribonuclease III DICER1 complexed with transactivation-responsive RNA-binding protein (TRBP, TARBP2) or protein activator of interferon-induced protein kinase (PACT, PRKRA). This provides approximately 22 nucleotide-long miRNA duplexes [27,28]. miRNA strands with unstable pairings at the 5′ end usually represent guide strands, and passenger strands with stable pairing are normally degraded [29]. The mature guide miRNA strand is bound by the ribonuclease argonaute 2 (AGO2), and miRNA-induced silencing complex (miRISC) binds to the 3′ UTR of the mRNA target by miRNA complementarity in the 2-8 nucleotide-long sequence. Near-perfect pairing leads to mRNA degradation due to steric hindrance, and partial complementation induces decreased expression by either mRNA removal or translation impairment, because the initiation site for the RNA-polymerase is blocked [30,31]. Several published studies, however, have indicated that miRNA binding to other regions in target mRNA contributes to mRNA translation maintenance. This indirect mechanism is mediated through miRNA interactions with AU-rich elements in the 3′ UTR region or by the binding sequences of regulatory ribonucleoproteins, both of which are located in target mRNAs. These interactions led to the recruitment of repressor protein complexes which release mRNAs from translational inhibition [32,33]. A further study demonstrated the initiation of transcription by direct binding of specific miRNAs to the 5′ UTRs of target mRNAs in the human foetal kidney and Wilms’ tumors [34]. Yet another study documented that miRNAs can also target promoter sequences and this results in gene expression initiation [35]. In addition, next generation sequencing (NSG) technology enables detection of many miRNAs isoforms (isomiRNAs) which originate from an miRNA locus. The isomiRNAs can be generated by imperfect cleavage during pre-miRNA processing and post-transcription modifications that affect the miRNA stability, subcellular location, and target selection. These physiological isomiRNAs also have multiple functions in miRNA biogenesis by modulating target recognition [36,37,38].

Single miRNAs can generally influence the expression of many mRNA targets and simultaneously regulate various biological processes. Each step in miRNA biogenesis in healthy cells from transcription to the production of mature miRNA controls many regulators. This was superbly demonstrated in animal cells. Disruptions to these control mechanisms and imbalances in the miRNA pool have been associated with numerous human diseases [39].

## 3. MicroRNA Functions in Cancer

The miRNAs in human cancers are differentially expressed compared to those in healthy cells, and the association between miRNA expression and cancer development was first described in chronic lymphocytic leukaemia. The chromosome 13q14 region and miR-15a/16a cluster in these patients was frequently deleted, thus indicating that these miRNAs were likely tumor suppressors [40]. 

Cancer-associated miRNAs are generally divided into two categories. The oncogenic miRNAs (oncomiRs) in the first category are usually highly expressed. They contribute to tumor progression and are important in the maintaining tumor phenotype. The second category contains tumor suppressive miRNAs (miRsupps) which inhibit tumorigenesis by regulating cell growth, apoptosis, immune-cell development and other cancer-associated events, and these are frequently down-regulated in various cancers. Some cancer-associated miRNAs are also known as context-dependent miRNA’s because they can act in a tissue-specific manner so that single miRNAs can have either oncogenic or tumor suppressive roles in different cancers. Examples of miRNAs with dual functions described by several authors include miR-17 (tumor suppressive in BC and oncogenic in B-cell lymphomas), miR-26 (tumor suppressive in hepatocellular carcinomas and oncogenic in gliomas and metastatic lung cancers) and miR-29 (tumor suppressive in lung tumors, cell lymphomas, and acute myeloid leukemias and oncogenic in B-cell chronic lymphocytic leukemia and BC) [41,42].

Recent research has also shown that miRNAs have a key function in cancer cell metastatic spread. These include invasion and migration association with the micro-environment and development of a poor prognostic phenotype. These miRNAs are termed metastamiRs, and they have been both up-regulated and down-regulated in different neoplasias, including BC [43,44]. While older studies documented that miRNA expression profiles in multiple human cancers exhibited global down-regulation of the miRNA pool compared to normal tissue miRNAs, different tumor types had varying miRNA expression profiles which could be utilised in discriminating the cancer type or tissue origin of poorly differentiated tumors [45,46].

The first large-scale bioinformatic analysis of human oncomiRs and miRsupps identified different function patterns, evolutionary rate, gene expression, chromosome distribution, molecule size, free energy, transcription factors, and targets. This indicates that oncomiRs more frequently cleave target mRNAs than miRsupps in human cancers. Furthermore, the oncomiR-encoding sequences were predominantly present in amplified chromosomal regions, unlike the miRsupp sequences which were more often located in deleted chromosomal regions [47]. While these “pioneer” results support evidence that oncomiRs in cancer patients are usually up-regulated and miRsupps deleted or down-regulated, the expression of several traditional oncomiRs in patient plasma samples have decreased during BC progression. These data indicate that the oncogenic or tumor suppressor characteristics of individual miRNAs cannot be strictly defined [48,49,50]. Current progress in molecular methodology has led to the application of miRNA microarrays and NSG in identifying miRNA series which more accurately discriminate the different cell types, including cancer cells. Moreover, the detection of many cancer-associated miRNA isoforms confirms that these isomiRNAs are important in miRNA-mRNA regulatory networks, and that altered isomiRNA expression profiles contribute to cancer development [37,51,52].

## 4. Destabilisation of microRNA Biogenesis in Cancer

Numerous changes in miRNA expression levels in human cancers are driven by dysregulation of miRNA biogenesis, and miRNA pool imbalance is due to up-regulation or down-regulation of miRNA-processing machinery components. The encoding genes of the major DROSHA, DGCR8, and DICER1 components are frequently up-regulated in many solid tumors which modify the global miRNA expression profile and contribute to the main attributes of cancer progression, such as increased cell proliferation, migration and invasion [53,54]. Studies have also shown that the *DROSHA* and *DICER1* gene down-regulation and consequent protein expression in many different tumors leads to decreased miRNA levels, and is clinically associated with invasion, metastasis, and poor patient survival [54,55,56].

Impairment of miRNA biogenesis is affected by both genetic and epigenetic alterations in miRNA regulation factors. The varying somatic and germline mutations in the *DROSHA*, *DGCR8, XPO5* and *DICER1* genes have been identified in the Wilms tumors (childhood kidney cancer) [57,58]. Further *DICER1* mutations were found in the pleuro-pulmonary blastoma (paediatric lung tumor) and also in non-epithelial OC [59,60]. In addition, heterozygous *XPO5*-inactivating mutations resulting in the impairment of pre-miRNA export from the nucleus to the cytoplasm were found in endometrial, colon, and gastric tumors with microsatellite instability [61]. The results from a very recent meta-analysis also identified two *DROSHA* polymorphisms and a *DGCR8* polymorphism with important roles in human tumorigenesis in both laryngeal cancer and BC [62]. In BC patients, the decreased mRNA expression of *DROSHA* and/or *DICER1* has been observed in 15% to 75.5%, and these levels were significantly associated with high grade tumors and a high Ki-67-induced cell proliferation index [63,64]. Other reports indicate that decreased *DICER1* mRNA levels were significantly associated with hormonal receptor status and the luminal A subtype, and that this decrease was predominantly noted in patients with metastatic disease [65]. Another study showed the gradual loss of *DICER1* protein expression in breast tissues during development of ductal carcinoma in situ (DCIS) and that the most significant reduction was found in metastatic malignant cells. This loss of DICER1 protein was especially observed in patients with decreased disease-free survival and in the more aggressive tumors characterised by higher grades and loss of the hormone receptor and BRCA1 DNA repair-associated (BRCA1) protein expression [66]. While decreased *DICER1* mRNA expression and increased *DROSHA* levels were identified in triple negative BC (TNBC) compared to the normal adjacent tissue, there were no differences in *DROSHA* expression between lymph node metastases (LNM) and primary tumors, but *DICER1* expression was significantly increased [67,68]. The combination of *DROSHA* up-regulation and *DICER1* down-regulation can initiate accumulation of primary miRNA transcripts and incomplete miRNA maturation, and these can contribute to cancer progression.

While no pathogenic mutations or epigenetic changes in the encoded genes of the two crucial DROSHA and DICER1 enzymes involved in miRNA regulation have been identified in breast tumors, there has been one polymorphism in each of the *DGCR8* and *DROSHA* genes established in a group of Chinese and African women, and these were significantly associated with BC risk [69,70]. Furthermore, in a case-control study of BC, one missense polymorphism and a high or high/middle methylation index in the *XPO5* gene were detected in blood DNA samples, and these were associated with an increased and a reduced risk of BC, respectively [71].

Three additional polymorphisms have been located in 14 genes functioning in miRNA biogenesis. These are in the *AGO1*, *AGO2*, and *DEAD-box helicase 5* (*p68, DDX5*) genes which correlate with BC risk [72]. This further indicates that genetic variants in genes regulating miRNA biogenesis could be extremely useful in evaluating BC development risk. In addition, the two miR-103/107 and miR-191/425 clusters targeting the *DICER1* gene influenced BC miRNA processing deregulation, and their up-regulation promoted BC tumor cell growth, invasion, and metastasis. Finally, it was further established that miR-103/107 contributed to the initiation of epithelial-to-mesenchymal transition (EMT) by down-regulating miR-200 [73,74].

## 5. MicroRNA Dysregulation in Invasive Breast Cancer

The key process required for BC cell spread to secondary organs is cancer cell invasion, and this can be mediated by identified cell interaction mechanisms such as EMT, collective invasion, and macrophage-cancer cell feedback loops. These involve multiple interactions between tumor cells and stromal cell sub-populations and proceed through soluble factor signaling, direct cell-cell adhesion, and extracellular matrix (ECM) re-modeling [75]. 

The specific breast cancer stem cell heterogeneous sub-populations of invasive cancer cells (BCSCs) have now been characterized, and they are proven capable of the self-renewal, differentiation, tumorigenesis, and chemoresistance essential for BC progression, cancer relapse, metastasis, and poor prognosis [76]. Compared to normal cells, they initiate the multiple changes in gene expression involved in invasion-associated pathways as a result of several mechanisms, including abnormal miRNA biogenesis.

### 5.1. MicroRNAs and Cell Adhesion

Cell-cell and cell-ECM adhesion maintenance is essential for normal cell and organism homeostasis and this is ensured by the multiple activities of cytoskeletal regulatory proteins, cell-cell adhesion molecules and ECM proteins [77]. The deregulation of adhesion-associated molecules, frequently influenced by aberrantly expressed miRNAs, enables cancer cell detachment and metastatic spread [78].

#### 5.1.1. MicroRNAs and Cytoskeletal Structure

Actin polymerisation and de-polymerisation in the highly dynamic cytoskeleton leads to significant changes in cell behavior, dependent on currently active cellular functions. These processes are regulated by the Ras homologue (Rho) superfamily of small GTPases [79]. Several miRNAs targeting Rho superfamily members have been identified in BC cells. For example, miR-155 directly inhibits the expression of RhoA protein [80], and miR-10b expression is induced by the Twist family basic helix-loop-helix transcription factor (TWIST) to initiate invasion indirectly by targeted suppression of homeobox D10 (HoxD10) and up-regulation of the pro-metastatic *RhoC* gene [81]. Another Rho superfamily member, the small GTPase cell division cycle 42 (CDC42), together with C-X-C motif chemokine receptor 4 (CXCR4), promoted cell invasion following down-regulation of its targeting miR-224 [82]. The oncogene *p-21 activated kinase 1* (*PAK1*) encodes the serine/threonine p21-activating kinase PAK1, a critical effector that associates with RhoGTPases during cytoskeletal re-organisation. In addition, the PAK1-encoding gene has been identified as a direct target of miR-494, and there is high PAK1 protein expression in BC samples resulting from miR-494 down-regulation. This could then contribute to the clonogenic activity, migration, and invasion observed in the BC cell lines [83]. Furthermore, the cytoskeletal protein tropomyosin 1 (TPM1) encoded gene is an accepted tumor suppressor. This is directly regulated by miR-21 [84]. miR-661 contributed to tight junction destabilization by targeting the encoded gene of two proteins. The first protein, nectin cell adhesion molecule-1 (Nectin-1), regulates cell-cell junctions and cytoskeletal organization, while the second phospholipid transferase STAR-related lipid transfer domain protein contains 10 (STARD10) functions in epithelial cell polarity. Further, the up-regulation of miR-661 in SNAI1-induced EMT cells enables cell invasion by suppressing epithelial markers. In BC samples, expression of the *STARD10* gene is highly associated with markers of luminal subtypes, but the negative correlation between its loss and markers of EMT-related, basal-like subtypes has been reported [85].

The connexin 43 (CX43) adhesion molecule mediates intracellular communication [86] and also influences cytoskeletal modification and cell migration, and this was found to be regulated by miR-200a and miR-206. Two studies have also reported that decreased miR-200a or miR-206 levels and increased *CX43* mRNA and protein levels are associated with cell proliferation, migration, and invasion capabilities. Moreover, higher mRNA *CX43* expression levels were found in BC discrete pulmonary and combined pulmonary and hepatic metastases than in the primary tumor [87,88]. The Wiskott–Aldrich syndrome protein family member 3 (WAVE3, WASF3) protein is a member of the WAVE actin cytoskeleton re-modelling family and this was highly expressed in BC, especially in the advanced stages. When the miR-200 cluster and miR-31 targeted *WAVE3* gene expression, there was a decrease in the levels of these miRNAs in BC cell lines, and the tumor tissues exhibited cytoskeletal alterations which contributed to an invasive phenotype [89,90]. In addition to this modifying effect, WAVE3 has a novel function in nuclear factor kappa B (NF-κB) signaling because it contributes to ECM degradation and invadopodia growth regulation [91].

The transmembrane cell-cell junction protein junctional adhesion molecule-A (JAM-A) participates in tight junctions and influences the cytoskeletal structure through its association with the actin-bundling protein facsin [92]. The loss of *JAM-A* gene targeting by miR-145 over-expression in several BC lines resulted in decreased cell motility and invasiveness [93]. While down-regulation of miR-145 expression in BC supports these results and could enable *JAM-A* gene sufficient expression to increase cancer cell motility [94], contrasting active BC cell migration was observed following *JAM-A* inhibition by up-regulated miR-495 [95].

#### 5.1.2. MicroRNAs and Cell-Cell Adhesion Junctions

Cell-cell adhesion junctions are the main protectors of tissue integrity, and cell-cell adhesion receptors enable initiation of many signaling transduction pathways [77]. Among the superfamily of cadherins, the transmembrane glycoprotein E-cadherin encoded by the *Cadherin 1* (*CDH1*) gene generates the fundamental adherens junctions between adjacent epithelial cells, and its cytoplasmic tail interacts with many intracellular proteins to mediate the association between E-cadherin and the actin cytoskeleton [96,97]. In many epithelial cancers, including BC, E-cadherin inactivation is considered a key event in the invasion-metastatic cascade, and E-cadherin immunostaining has proven useful in discriminating breast lobular lesions from ductal lesions in indecisive histological result [98]. 

In addition to being affected by several *CDH1* somatic mutations and loss of heterozygosity (predominantly in invasive lobular BC) and promoter methylation [99,100,101], the *CDH1* gene was found to be directly inhibited by miR-9. The up-regulation of this miRNA was associated with invasiveness and metastatic status [102]. While a decreased miR-9 level was found in benign tumors compared to normal tissues, it was increased in malignant vs. benign tumors, but not equal to the level expressed in normal tissues. These results demonstrate the dynamic changes in miR-9 and its multiple different functions during tumor progression [103]. In addition, *CDH1* silencing indirectly regulated other miRNAs by targeting several essential transcription factors which contribute to cancer cell EMT. This relationship is reviewed in subsequent Section 5.3.

The CUB domain-containing protein 1 (CDCP1) transmembrane glycoprotein has been defined in BC cell lines as a regulator of cell adhesion and motility [104]. This protein contributes to the disruption of adherens junctions and is found widely expressed in human epithelial cancers and associated with advanced stages and poor patient survival. Increased *CDCP1* gene expression could be a consequence of down-regulation of the targeting miR-198 [105,106].

#### 5.1.3. MicroRNAs and Cell-ECM Interactions

The attenuation of cell-ECM interactions is a further prerequisite for cancer cell detachment. The primary receptors of ECM proteins present on the cancer cell surface, integrin α/β heterodimers, mediate bidirectional signaling across the cell membrane. This enables regulation of many cellular processes, such as cell proliferation, differentiation, survival, adhesion, and motility and integrin deregulation, thereby contributing to cancer invasiveness and metastasis [107]. miR-31 appears to be a key regulator of integrins through its targeting of several α subunit partners of β1 (α2, α5, and αV) and β3 integrins [108]. Furthermore, several studies have investigated the regulation by miRNA of individual integrins. Decrease in *integrin α2* (*ITGA2*) mRNA in BC cells was caused by miR-373 targeting associated with the degradation of cell-cell interactions, depolymerisation of F-actin fibres and cell migration integration. Moreover, significantly decreased ITGA2 protein levels and increased miR-373 expression were found in BC, predominantly in patients with LNM, compared to those in adjacent non-malignant tissues [109]. In contrast, a further in vitro study showed that the over-expression of miR-142-3p affected several cytoskeletal structure and cell motility genes, including *Wiskott–Aldrich Syndrome Like* (*WASL*) and *ITGAV* which inhibits BC cell invasiveness [110]. Moreover, one of the β1 family integrins (α3β1) indirectly affected BC invasion by activating Rac1/PAK1 pathway signaling, mitogen-activated protein kinase (MAPK), c-Jun NH2-terminal kinase (JNK), and PAK1 [111]. 

Two kinases, PAK1 and focal adhesion kinase (FAK), were shown to be directly targeted by HoXD10-dependent miR-7, and combined down-regulation of this miRNA, PAK1, and FAK protein up-regulation was associated with a more invasive phenotype. More evident decrease in miR-7 was also observed in metastatic BC patients [112,113]. Furthermore, miR221/222 were demonstrated as the main regulators of BC cell proliferation and invasion by targeting the *signal transducer and activator of transcription 5A* (*STAT5A*), *disintegrin and metalloproteinase domain 17* (*ADAM17*) and *integrin β4 (ITGB4)* genes; *ITGB4* encodes an adhesion molecule that interacts with laminin receptors. Although the authors did not confirm inverse correlation between miR-221/222 and *ITGB4* expression, ITGB4 protein expression and miR-221/222 down-regulation in luminal BC was observed in patients with poorly differentiated G3 tumors [114].

The ADAM multidomain proteins, which have two main disintegrin and metalloprotease domains, are involved in proteolysis and cell adhesion and are predominantly located on the cell membrane. Proteolytically active ADAMs function in ECM re-modeling by shedding variable substrates, such as adhesion ligands, growth factors and their receptors and various cytokines. Expression changes in ADAMs, predominantly in ADAM9, 10, 12, 15, and 17, were associated with cancer progression [115]. There, transcription of the ADAM9-encoding gene was found to be targeted by miR-33a, miR-126, and miR-154. These miRNAs inhibit the proliferation, invasion, and migration of cancer cells and the gradual decrease in their levels in tumorigenesis was observed from early stages to non-metastatic and LNM-positive BCs compared to normal breast tissues [116,117,118]. 

In contrast, ADAM8 regulates several miRNAs including miR-720. The disintegrin and cysteine-rich domains of this protein activated miR-720 expression through the extracellular signal-regulated kinase (ERK) signaling cascade which induces aggressive phenotypes in TNBC cells. Moreover, increased miR-720 levels were present in the serum of TNBC patients with the high expression of ADAM8 [119]. ADAM proteins without metalloproteinase domains are proteolytically inactive, but they interact with integrins through their disintegrin domains. This affects cell adhesion [120] and changes in these proteins contribute to cancer invasion. For example, *ADAM23* is considered a tumor suppressor and it is epigenetically inactivated by promoter methylation in BC patients [101,121,122]. No miRNA regulating its encoding gene has yet been identified; however, we previously published that *ADAM23* most likely participates in the spread of mesenchymal circulating tumor cells [123]. 

### 5.2. MicroRNAs and the Tumor Micro-Environment

Many studies have shown that changes in the micro-environment, consisting of different types of stromal cells such as fibroblasts, endothelial cells, adipocytes, and immune cells combined with ECM components has an essential role in cancer progression [124]. Aberrantly expressed stromal miRNAs can regulate intercellular crosstalk within the TM [125]. Cancer-associated fibroblasts (CAFs) produce many cytokines and chemokines with important functions in invasion and metastasis. In addition, the interaction between fibroblast-derived C-X-C motif chemokine ligand 12 (CXCL12, SDF1) and endothelial cells was recently reported to enhance tumor cell intravasation by increasing vascular permeability [126]. The *CXCL12* gene is directly targeted by miR-126 and miR-126* and results in the suppression of the CC-motif chemokine ligand 2 (CCl2) produced by cancer cells in a CXCL12-dependent manner. The authors documented that miR-126 and miR-126* can modify the TM and they demonstrated the inhibition of BC metastasis in a mouse xenograft model through the miR-126- and miR-126*-dependent suppression of mesenchymal stem cells and inflammatory monocyte recruitment in the tumor stroma environment [127]. The stromal fibroblast-produced chemokine CXCL12 primarily binds to the highly expressed CXCR4 receptor on cancer cell surfaces, resulting in the induction of intracellular signaling which lead to tumor progression, angiogenesis, metastasis, and survival [128]. A further study reported that miR-494 significantly inhibited the CXCR4 protein alone and that it was down-regulated in BC cells. This miRNA also contributed to the inhibition of BC development through the CXCR4-dependent Wnt/β-catenin signaling pathway [129]. 

Further, miR-320 was detected in both animal models and BC patient samples as a downstream regulator of *phosphatase and tensin homologue* (*PTEN*) gene in stromal fibroblasts. The *PTEN* gene affected the mRNA expression profiles in neighboring endothelial and epithelial cells in breast tissue. In addition, miR-320 down-regulation in BC and the up-regulation of its direct *ETS proto-oncogene 2* (*ETS2*) target were crucial in the loss of *PTEN* which promoted tumor invasiveness and angiogenesis by micro-environmental modification [130]. Further authors found that stromal miR-200 cluster members (miR-200a, miR-200b, miR-200c, and miR-141) directly mediated the re-programming of normal fibroblasts into CAF. The down-regulation of these miRNAs enabled high expression of their target genes *friend leukemia integration 1* (*FLI1*) and *transcription factor 12* (*TCF12*) which functioned in the development and differentiation of cells and resulted in ECM re-modeling and BC cell invasion and metastasis [131].

In addition to stromal cell proteins, aberrantly expressed miRNAs modulated the expression of basic ECM proteins, such as collagens, laminins, and fibronectin. It was observed that these acted as ligands for integrin extracellular domains in several types of cancer [77,78]. The up-regulation of *laminin subunit alpha 4* (*LAMA4*) mRNA and protein expression in TNBC and decreased miR-539 targeting have also been established [132]. Finally, an association between metastatic suppression and miR-335 expression targeting the ECM component tenascin C encoding gene (*TNC*) was observed by examining other structural ECM proteins including glycosaminoglycans, proteoglycans, and matricellular proteins [124], and the loss of miR-335 was detected in BC patients with poor prognosis [133].

Furthermore, several ECM re-modeling enzymes directly influenced ECM function and biomechanical properties that promoted the detachment of cancer cells from primary tumors. Collagen proteolysis in the basement membrane was induced by the high expression of matrix metalloproteinases [124]. Among them, matrix metallopeptidase 14 (MMP14), which is located on the surfaces of BC cells, was shown to be active in collective migration [134], and the up-regulation of MMP14 and the down-regulation of the targeting of miR-181a-5p were more pronounced in the invasive front of breast tumors than in adjacent normal tissue [135]. On the other hand, the miR-21 up-regulation, which was found predominantly in patients with LNM associated with a decrease in tissue inhibitor of metalloproteinases 3 (TIMP3) protein, promoted ECM degradation and cancer cell invasion [136].

The additional urokinase plasminogen activator (uPA) enzyme was found to catalyse the transformation of plasminogen to active plasmin to initiate ECM and basement membrane disintegration through the activating several pro-MMPs [137]. The up-regulation of uPA expression in metastatic BC and the negative association between the expression of miR-193b and uPA protein reflected the role of this miRNA in BC invasiveness [138]. However, lower levels of mature miR-193a, miR-193b, and miR-181a but not their primary targeted miRNA transcripts were found in BC cells that highly expressed uPA in association with the decreased expression of DROSHA and DGCR8, which illustrates the effect of impaired miRNA biogenesis on uPA up-regulation [139].

Transcription factor gata-binding protein 3 (GATA3) has a quite complex function in micro-environmental re-modeling and metastasis inhibition through its regulation of miR-29b expression, and down-regulation of miR-29b in GATA3-expressing luminal breast cancers resulted in metastatic development and a mesenchymal phenotype in cancer cells. The authors proposed that miR-29b inhibited metastasis by targeting several pro-metastatic regulator genes involved in angiogenesis, collagen re-modeling and proteolysis, such as *vascular endothelial growth factor A* (*VEGFA*), *angiopoietin-like 4* (*ANGPTL4*), *platelet-derived growth factor* (*PDGF*), *lysyl oxidase (LOX)* and *MMP9*, and by targeting *ITGA6, ITGB1* and *transforming growth factor beta (TGF-β)*, to influence differentiation and epithelial plasticity [140].

### 5.3. MicroRNAs in Epithelial-Mesenchymal Transition

EMT and the reverse process, mesenchymal-epithelial transition (MET), are active in many physiological processes, including embryo implantation, embryogenesis and organ development. Under pathological conditions, these mechanisms participate in tissue regeneration, organ fibrosis, cancer progression, and metastasis development. During the EMT process, epithelial cancer cells lose their polarity, cell-cell contacts, and cell-basement membrane interactions and acquire a mesenchymal cell phenotype, which is characterized by enhanced migratory capacity, invasiveness, and increased resistance to apoptosis [141]. The EMT program is initiated by numerous extracellular signals and transcription factors, including TGF-β, Notch receptor and Wnt/β-catenin signaling pathways and growth factors that bind tyrosine kinase receptors, and these all frequently act in a sequential manner [142,143]. The multi-functional cytokine TGF-β is a potent inducer of EMT, and increased TGF-β levels were found in the plasma and invasive fronts of tumors in BC patients and these correlated with the presence of LNM [144,145]. Recently, the mechanisms of TGF-β1-induced EMT were described and were shown to promote the chemotaxis-mediated migration of BC cells through the lymphatic vessels [146]. Other authors found that miR-10b and miR-23a were directly regulated by TGF-β1 and that the up-regulated expression of these two miRNAs was associated with aggressive BC. Moreover, miR-23a directly targeted and inhibited *CDH1* gene, which activated Wnt/β-catenin signaling. These results indicate that both miR-10b and miR-23a contribute to TGF-β1-induced EMT and tumor metastasis in BC cells and patient tissues [147,148]. Furthermore, hyperactivated Wnt/β-catenin signaling was shown to drive EMT and metastasis in metastatic BC cells. In these cells, the ectopic overexpression of miR-374a was observed along with the simultaneous suppression of its direct targets *WNT inhibitory factor 1* (*WIF1*), *PTEN* and *WNT family member 5A (WNT5A)*, all of which are negative regulators of Wnt/β-catenin signaling. Significantly up-regulated miR-374a levels were observed in BC patients with distant metastases in association with poor metastasis-free survival [149].

The hallmark of EMT induction is the loss of adherent junctions through the inhibition of the main cell-cell adhesion molecule E-cadherin, which is encoded by the *CDH1* gene [150]. The transcription factors snail family transcriptional repressor 1 (SNAIL1), SNAIL2/SLUG, zinc finger E-box binding homeobox 1 (ZEB1), ZEB2, and TWIST1 (encoded by the *SNAI1*, *SNAI2*, *ZEB1*, *ZEB2*, and *TWIST1* genes) were shown to be important inducers of the EMT process. These repress E-cadherin expression by binding to the E-box elements in the promoter region of the *CDH1* gene [151]. However, both E-cadherin alone and EMT inducers are regulated by several miRNAs.

A well-investigated cluster implicated in EMT regulation is miR-200. This has five members grouped into two independent transcriptional units where miR-200b, miR-200a, and miR-429 are clustered in an intergenic region of chromosome 1, and miR-200c and miR-141 are located on chromosome 12 in an intron of a non-coding RNA [152]. Within 70 TNBC breast tumors, additional authors found that human epidermal growth factor receptor 2-positive (HER2+) patients with predominantly metaplastic cancers exhibited decreased expression of the miR-200 cluster compared to those with oestrogen receptor-positive (ER+) tumors. Decreased expression of all five miR-200 cluster members was observed in metaplastic BC and in vitro models of spontaneous EMT. This was combined with increased expression of the EMT-transcriptional inducers, *SNAI1*, *SNAI2*, *ZEB1*, and *ZEB2* genes, and the hypermethylation of the miR-200c/141 locus [152]. 

Several studies have investigated the role of individual miR-200 cluster members in EMT. There, the Kindlin family proteins were previously defined as regulators of integrin functioning. They exhibited different expression profiles and participated in many biological processes, including cell adhesion and migration [153]. The expression of Kindlin-2 was associated with the BC metastatic phenotype in human and mouse BC cells, and the direct targeting and inactivation of its encoding gene, *fermitin family member 2* (*FERMT2*), by miR-200b resulted in the inhibition of EMT and metastasis [154]. In further study described tumor protein p53 binding protein 1 (TP53BP1, 53BP1) as a novel negative regulator of EMT through the miR-200b/429-mediated down-regulation of *ZEB1*. Although *53BP1* expression positively correlated with both miR-200b and miR-429 expression and negatively correlated with *ZEB1* gene expression in 18 BC samples, in vitro experiments failed to show direct interaction between *53BP1* and these two miRNAs [155]. Recently, the actin interacting proteins SH3 and PX domains 2A (TKS5, SH3PXD2A) and myosin light chain kinase (MYLK) were identified as novel targets of miR-200c. The authors showed that the *ZEB1*/miR-200c feedback loop controlled the invasion of tumor cells. The simultaneous activation of EMT and invadopodia formation was observed in BC cell lines and patient samples, and this was associated with the co-expression of *ZEB1* and both the *TKS5* and *MYLK* genes and down-regulated targeting by miR-200c [156]. It was further discovered that p53 induces miR-30a expression in TNBC patients by binding to the *MIR30A* promoter, and this miRNA directly targets *ZEB2* expression. In addition, the decreased expression of miR-30a in BC was found to correlate with p53 deficiency, LNM and poor prognosis. This study provided evidence that tumor aggressiveness is controlled through the p53/miR-30a/ZEB2 axis, which could subsequently affect miR-200c expression [157].

Another important EMT related cluster, DLK1-DIO3, was shown to comprise seven tumor-suppressing miRNAs (miR-300, -382, -494, -495, -539, -543, and -544) located on chromosome 14. These miRNAs target encoding genes of well-known EMT activators, such as TWIST1, ZEB1/2, and B lymphoma Mo-MLV insertion region 1 homologue (BMI1), and they were down-regulated in BC cells with EMT and also in invasive ductal BC samples. In vitro experiments showed in detail that miR-300, miR-539, and miR-543 targeted *TWIST1* gene and that miR-300, miR-494, miR-495, and miR-544 inhibited *BMI1* expression. It was then further identified that miR-494 and miR-539 repressed the *ZEB1* gene. Moreover, the up-regulation of miR-300, miR-494, miR-539, and miR-543 were significantly associated with increased expression of specific miR-200 cluster members; this could result from the inhibition of *TWIST1* which was considered a miR-200 repressor through its binding to the promoter region of this cluster [158].

In addition to the abovementioned clusters, the other main EMT inducers are regulated by other miRNAs. The SNAIL1 protein-dependent activation of EMT was observed in p53 loss-of-function cancer cell lines, including BC, because of a decrease in the levels of miR-34 which is a direct regulator of *SNAI1*. These results demonstrated the interaction between p53, miR-34, and SNAIL1 in the EMT programme [159]. Other authors observed decreased miR-124 levels combined with simultaneous up-regulation of the expression of the targeted *SNAI2* gene in BC cell lines and patients [160]. In a further BC in vitro study, it was observed that miR-203 and miR-200 cluster member expression decreased in a time-dependent manner during *SNAI1*-induced EMT. Moreover, miR-203-inhibited endogenous *SNAI1* formed a double-negative miR-203/*SNAI1* feedback loop and as with the known miR200/*ZEB1* feedback loop, this could function as an EMT-inducing core network [161]. In a miRNA microarray study, 21, 47, and 107 different miRNA expression profiles were found in DCIS, invasive, and metastatic BC samples compared to normal tissues, and miR-205 down-regulation was associated with a metastatic phenotype [162]. miR-205 was shown to directly target encoding genes of the ZEB1 and ZEB2 transcription factors which initiate EMT processes [163]. Other authors showed a direct relationship between the SNAIL2/SLUG transcription factor and miR-221 in BC cell lines. The expression of miR-221 was found to be partially SNAIL2-dependent, and the inhibition of cell migration was associated with SNAIL2 silencing and the maintenance of miR-221 expression [164]. Moreover, miR-221 directly targeted the open reading frame of *CDH1*, resulting in E-cadherin suppression. In this study, a novel mechanism involving the post-transcriptional inhibition of E-cadherin by SNAIL2-promoted miR-221 overexpression was demonstrated [165]. Both miR-221 and miR-222 were described as specific miRNAs that promoted a basal-like BC subtype, and their increased expression resulted in the down-regulation of epithelial genes and the up-regulation of mesenchymal-specific genes that contributed to invasiveness and increased cancer cell migration. Additionally, miR-221/222 indirectly reduced the expression of E-cadherin by targeting of the transcriptional repressor gata binding 1 encoding gene (*TRPS1*), which directly represses *ZEB2* transcription [166]. Compared to the main EMT-associated transcription factors, the miRNA regulation of TWIST1 expression in BC has been less frequently investigated. The *TWIST1* gene was targeted by miR-720, and the down-regulation of this miRNA was associated with the presence of lymph node metastasis [167].

Furthermore, miR-506 was predicted to target other EMT-participating genes, and the down-regulation of miR-506 was observed in BC tissues [168]. The over-expression of miR-506 led to the inhibition of TGF-β-induced EMT and prevented cell adhesion, invasion, and migration through the down-regulation of the mesenchymal genes such as *SNAI2*, *vimentin* (*VIM*), and the *CD151 molecule (raph blood group)* (*CD151*). Moreover, NF-κB suppressed miR-506 transcription by binding to its upstream promoter region [169]. Another EMT inhibitor, miR-153, was found to be down-regulated in metastatic BC cell lines and BC samples. Normally expressed miR-153 directly targeted the *metadherin (MTDH)* gene, an inductor of EMT, which resulted in the miR-153-induced suppression of the migration and invasion of BC cells [170]. Furthermore, miR-375 targeted the short stature homeobox 2 (SHOX2) transcription factor encoded gene which contributed to the activation of the TGF-β signaling network and EMT induction. The authors analyzed microarray data for BC gene expression from the Gene Expression Omnibus database and revealed that the loss of miR-375 expression and the gain of *SHOX2* expression was associated with poor survival in BC patients; and this illustrates the role of *SHOX2* in BC progression [171].

### 5.4. MicroRNAs and Cancer Stemness

The development of BCSCs, a small subset of cancer cells with stem cell-like properties, is closely linked to the successful completion of the invasion-metastasis cascade by the dissemination of cancer cells and is driven by EMT induction [172,173]. BCSCs stimulate EMT by releasing TGF-β and expressing specific miRNAs which target genes and the signaling pathways important for the regulation of stem cell properties, such as self-renewal and differentiation [174,175].

The balance between self-renewal and differentiation is an important characteristic of BCSCs, and multiple miRNAs participate in regulating this equilibrium. However, the critically important for the stemness maintenance of human BCSCs is the suppression of the three miRNA miR-200, miR-183, and let-7 clusters, and the up-regulation of miR-221 [176,177,178,179,180]. Meanwhile, down-regulation of the miR-200 cluster results in the increased expression of polycomb repressor complex proteins, such as BMI1 and suppressor of zeste 12 homologue (SUZ12) which are known regulators of stem cell self-renewal and pluripotency and enhance the development of the abovementioned features of BCSCs [176,181]. SUZ12 controls the expression of E-cadherin and the miR-200/SUZ12/E-cadherin axis, and this is crucial in maintaining the BCSC phenotype and regulating BC metastasis. The over-expression of miR-200b blocks BCSC formation by inhibiting SUZ12 and prevents the repression of E-cadherin, thus regulating tumor growth and invasiveness [181]. The interactions between miR-200 cluster members and transcriptional factors such as ZEB1 and ZEB2 can also inhibit the transcription of the entire miR-200 cluster [182,183], and specific protein 1 (Sp1) and p53 binding has been shown to lead to the activation of miR-200b/200a/429, miR-200c, and miR-183 transcription [184,185].

Furthermore, the association between the miR-200 cluster, miR-22, and ZEB1/ZEB2 has an important function in stemness regulation and EMT. MiR-22 exerts its metastatic potential through the direct targeting of encoding genes of the ten eleven translocation (TET) family of methylcytosine dioxygenases, thereby inhibiting the demethylation of the miR-200 cluster promoters and suppressing its expression. The over-expression of miR-22 is correlated with poor clinical outcomes and the silencing of the TET-miR-200 cluster axis in patients [186].

The miR let-7 regulates the key BCSC features, including self-renewal, multipotent differentiation, and the ability to form tumors, and authors have indicated that miR let-7 expression is reduced in BCSCs compared to non-stem cancer cells in primary tumors and cancer cell lines [178]. In contrast, increased let-7 levels result in reduced protein expression of the Harvey rat sarcoma viral oncogene homologue (H-RAS) and the high mobility group AT-hook 2 (HMGA2) which are known let-7 targets [187,188]. These genes encode DNA-binding proteins implicated in mesenchymal cell differentiation and tumor formation, and they are related to the self-renewal and pluripotent potential of stem cells. Moreover, while H-RAS silencing in a BCSC-enriched cell line reduced self-renewal with little effect on differentiation, HMGA2 silencing enhanced differentiation but not self-renewal [178]. Similarly, a reduction in miR-30 expression and the subsequent up-regulation of its direct gene target, *ubiquitin-conjugating enzyme 9* (*UBC9*), helped maintain the self-renewal capability of BCSCs. Ubc9 is essential for the sumoylation of numerous proteins related to the self-renewal process [189].

The, octamer-binding transcription factor 4 (OCT4), Sry-related high-mobility box 2 (SOX2), and homeobox transcription factor (NANOG) are the main transcription factors. These are known as the master regulators of stem cell pluripotency and are responsible for maintaining the undifferentiated state of embryonic stem cells (ESCs). These three factors bind to their own promoters as well as the promoters of the genes encoding the two other factors, and this auto-regulation enhances the stability of gene expression and facilitates the maintenance of the pluripotent state [190]. They are also directly epigenetically regulated by the binding of ESC-specific miRNAs to their promoter regions [191] and also at the post-transcriptional level by the progressive removal of DNA and H3K9 methylation [192] during the process of ESC generation [190]. As mentioned above, the over-expression of miR-221 contributes to stemness maintenance in BC. This miRNA operates as an oncomiR by targeting and repressing the *DNMT3B* gene, and this leads to extensive changes in the DNA methylation of several promoter regions, including the *NANOG* and *OCT 3/4* promoter regions. Decreases in the levels of these two stem cell pluripotency regulators were observed in BCSCs compared to those in differentiated cells [180], and conversely, miR-590-5p and miR-140 acted as tumor suppressors and inhibited stemness by targeting *SOX2* gene. This led to a decrease in the BCSC population [193,194].

## 6. Epigenetic Regulation of microRNA Expression

The aberrant expression of numerous genes, which results in their inefficient function, is a typical characteristic of tumor tissues. In cancers, changes in expression profiles can be induced by the accumulation of genetic alterations and epigenetic events, including the aberrant hyper- and hypo-methylation of DNA and changes in histone modifications following the re-modeling of the chromatin structure [195]. As mentioned above, miRNAs can operate as oncogenes, tumor suppressors, modulators of metastatic spread and regulators of cancer cell stemness. The dysregulation of miRNA expression in cancer is caused genetically or through epigenetic mechanisms, most frequently by the methylation of promoter miRNA by its own transcriptional units or those of host genes where the miRNA sequences are located [196,197].

Epigenetic inactivation was widely investigated in BC-associated miRNAs with tumor suppressor function and also those which mediate the inhibition of cell proliferation and EMT [198]. In addition, promoter methylation was identified in several miRNA genes that participate in the invasive processes reviewed in the previous sections. In miR-200 cluster members, which are considered to be the main EMT regulators, DNA methylation silencing was found only in the transcriptional unit miR-200c/141, and not in miR-200b/200a/429, despite the presence of CpG islands in both transcription start regions [152,199]. The direct association between the methylation of miR-200c/141 and the increased expression of the *SNAI1*, *SNAI2*, *ZEB1*, and *ZEB2* target genes in invasive BC reflected the critical role of miRNA epigenetic regulation in EMT processes [152].

Similarly, seven miRNAs (miR-300, -382, -494, -495, -539, -543, and -544) clustered in the DLK1-DIO3 region were simultaneously inactivated by the hyper-methylation of upstream promoter elements following activation of the EMT programme, predominantly within the TWIST1 protein signaling network [158]. Furthermore, miR-203 was previously found to be a regulator of the EMT-associated gene *SNAI1* [161], and miR-203 *SNAI2* targeting has also been observed in another study [200]. Moreover, the epigenetic silencing of miR-203 was observed in metastatic BC cells, and the loss of miR-203 expression contributed to EMT processes and cancer stem cell development [200,201]. EMT was also initiated as a consequence of the more complex epigenetic regulation of miR-205. The chromatin-modifying polycomb group ring finger 2 (Mel-18, PCGF2) protein affected miR-205 expression by inhibiting the DNA methyltransferase-mediated DNA methylation of the *MIR205* host gene promoter. This led to a decrease targeting of the *ZEB1* and *ZEB2* genes by actively transcribed miR-205. On the other hand, the loss of Mel-18 increased *ZEB1* and *ZEB2* expression and the invasion and migration of metastatic BC cell lines [163]. An opposite effect on miR-205 expression resulted from Erb-B2 receptor tyrosine kinase 2 (ErbB2) signaling which down-regulated miR-205 through the Ras/Raf/MEK/ERK pathway, and this influenced DNA methyltransferase activity and consequently led to the hyper-methylation of the *MIR205* host gene [202].

In another study, the down-regulation of miR-34c was found in breast tumor initiating cells with stem cell features that enabled self-renewal, EMT and cell migration as a consequence of the expression of *NOTCH4* which is a miR-34c target gene. The hyper-methylation of a single CpG site was identified in the promoter region of this miRNA and this caused miR-34c transcription inhibition by reduced Sp1 DNA binding activity [203]. As described in previous Section 5.1.1 and Section 5.1.3, miR-31 expression was associated with cytoskeletal re-modeling and cell-ECM interactions through integrin regulation [90,108]. The authors recently demonstrated the importance of miR-31 inactivation in BC invasiveness by promoter hyper-methylation in the *LOC554202* miR-31 host gene. This activity was predominantly noted in very aggressive TNBC cell lines of the basal subtype [204]. Similarly, miR-126/miR-126* was down-regulated by promoter methylation of its *EGF-like domain multiple 7* (*EGFL7*) host gene in cancer cells. Low levels of miR-126/miR-126* prevented mesenchymal stem cell and inflammatory monocyte recruitment in the TM, thus contributing to BC metastasis [127].

The miR-335 is defined as a metastatic suppressor, and although it is frequently silenced in human BC by genetic deletion, it was inactivated by promoter hyper-methylation in metastatic derivatives isolated from malignant cell populations from several different BC patients [205]. Other authors investigated the methylation profiles of three genomic loci of miR-124a in freshly frozen BC samples in greater detail and they identified correlation between DNMT3B protein over-expression and miR-124a-3 hyper-methylation. The simultaneous methylation of the miR-124a-1, miR-124a-2, and miR-124a-3 loci was associated with LNM and a high mitotic score in cancer tissues [206].

A great degree of epigenetic regulation is mediated by miR-221 which can be considered a key deregulator of DNA methylation in BC. This miRNA was shown to directly target the main de novo methyltransferase DNMT3B-encoding gene, which could lead to extensive changes in DNA methylation profiles, but this has only currently been observed in association with BC stemness [180].

Different kinds of epigenetic events associated with BC invasiveness are caused by histone methylation deregulation. In BC cell lines, miR-708 directly targeted the encoding gene of the important epigenetic regulator lysine-specific demethylase 1 (LSD1) which specifically demethylates mono- and dimethylated lysine 4 and lysine 9 in histone 3. The inhibition of miR-708 was associated with increased cell proliferation and invasion, but *LSD1* overexpression could counteract these effects of miR-708 [207]. Further authors showed that miR-708 suppression in metastatic BC was regulated by the polycomb repressor complex 2 (PRC2)-induced trimethylation of lysine 27 in histone 3 (H3K27). Moreover, the gradual decreasing of the miR-708-targeted *neuronatin* (*NNAT*) gene during breast metastatic progression enabled over-expression of neuronatin which regulates ion channels. Increased intracellular calcium levels can initiate cell migration and metastasis development [208]. 

**Figure 1:**
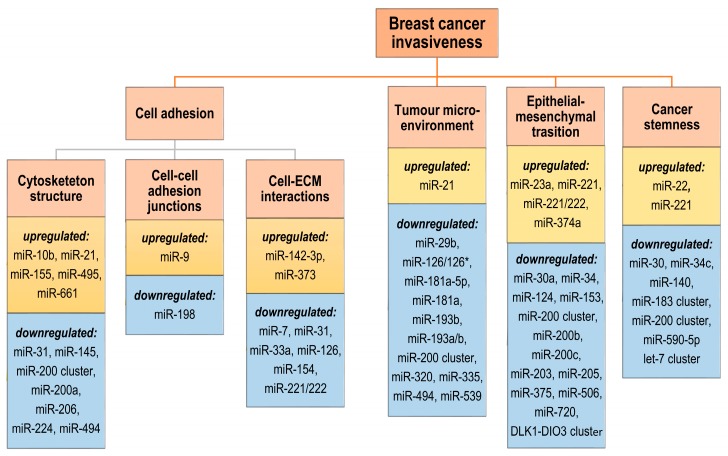
Up-regulated and down-regulated microRNA expression profiles in specific processes associated with breast cancer invasiveness and stemness. ECM, extracellular matrix. Note: miR-126*, the partner to miR-126 that is derived from the same transcript, had a similar expression pattern [127].

In summary, miRNAs associated with specific invasive processes and cancer cell stemness in BC and their changes in expression, types of epigenetic regulation, target genes, and participation in invasion-metastasis cascade steps are presented in Table 1. The up-regulated and down-regulated miRNAs are divided according to the individual processes involved in BC invasiveness in Figure 1.

## 7. Multifunctional microRNAs in Invasive Processes 

**Figure 2:**
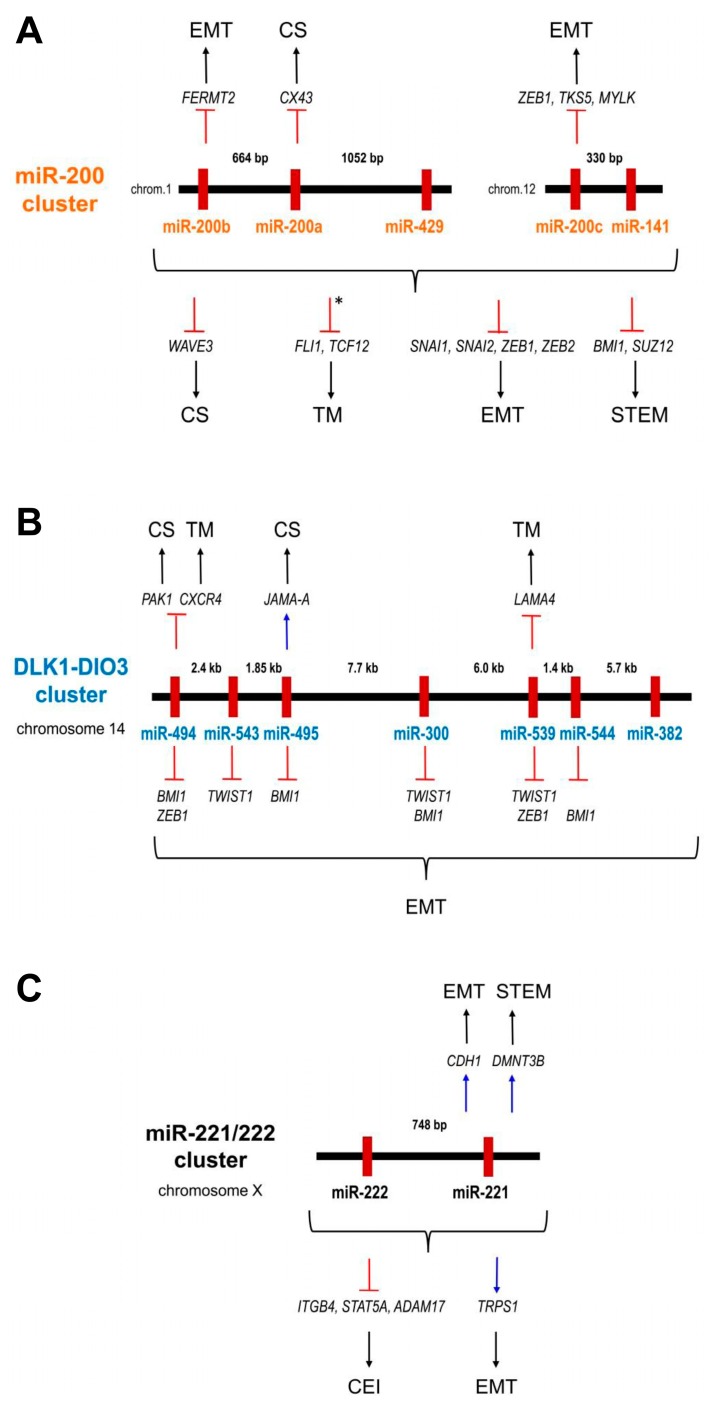
Regulation activities of miRNAs located in miR-200 (**A**), DLK1-DIO3 (**B**), and miR221/222 (**C**) clusters. Down- or up-regulation of these miRNAs influences the expression of their target genes which activate the specific events initiating BC invasiveness and stemness. Red T, inhibition of target gene expression; blue arrow, expression of target gene enablement; black arrow, involvement of target genes in specific processes associated with BC invasiveness and stemness. Abbreviations: CS, cytoskeleton structure; CEI, cell-extracellular matrix interactions; TM, tumor micro-environment; EMT, epithelial-to-mesenchymal transition; STEM, cancer cell stemness. Note: *miRs of miR-200 cluster without miR-429.

It is generally accepted that individual miRNAs can regulate many genes involved in different biological mechanisms. Therefore, some of the abovementioned miRNAs function simultaneously in several invasive processes. Multiple activities have been documented for miR-200, DLK1-DIO3, and miR221/222 clusters in normal cells (Figure 2A–C). Down-regulation or up-regulation of these miRNAs influence the expression of their target genes which results in initiation of previously summarised specific processes related to BC invasiveness and stemness. 

Members of the miR-200 cluster were found to be down-regulated in association with defects in cytoskeletal structure, TM, EMT modification, and cancer stemness. This down-regulation enabled increased expression of the cytoskeletal re-modeling gene *WAVE3*, followed by the *FLI1* and *TCF12* genes which contributed to ECM alterations, and finally the expression of both the *SNAI1*, *SNAI2*, *ZEB1*, and *ZEB2* EMT genes and the *BMI1* and *SUZ12* stem cell self-renewal and pluripotency regulators [89,131,152,176,181]. In addition, single miR-200 cluster members as miR-200a contributed to the cytoskeletal changes by increased expression of *CX43*, and miR-200b together with miR-200c promoted EMT initiation through up-regulation of *FERMT2* and *ZEB1*, *TKS5* and *MYLK* genes, respectively [87,154,156].

While the down-regulation of miRNAs from the DLK1-DIO3 cluster predominantly affected EMT reviewed in Section 5.3 [158], the loss of miR-494 contributed to cytoskeletal and micro-environmental re-organization by influencing the expression of the *PAK1* oncogene and the *CXCR4* chemokine receptor [83,129]. The noted TM changes were due to increased expression of laminin subunit *LAMA4* gene caused by down-regulation of miR-539 [132]. In contrast, increased miR-495 activated the cytoskeletal changes by inhibiting the JAM-A junctional adhesion molecule [95]. 

While decreased miR-221/222 influenced the cell-ECM interaction by deregulating adhesion and proteolytic molecules (Section 5.1.3. [114]), their increased expression promoted EMT induction by the direct or indirect inhibition of the *CDH1* gene. This contributed to the cancer stemness by targeting *DNMT3B* which then enabled the *NANOG* and *OCT 3/4* gene re-expression [165,166,180]. 

Several individual miRNAs contributed to the regulation of different combinations of invasive processes. For example, the up-regulation of miR-21 affected cytoskeletal and TM re-organization [84,136], and the down-regulation of other miRNAs, including miR-30a, miR-31, and miR-34c influenced several processes. Further, miR-30a and miR-34c promoted EMT and stemness, and miR-31 modified cytoskeletal and cell-ECM interactions, and miR-126 and miR-126/126* affected cell-ECM interactions and TM, respectively [90,108,117,127,157,159,189,203]. The different functions of miR-720 in breast tumorigenesis were indicated by its increased expression in the serum of ADAM8-expressing TNBC patients, and its down-regulation in primary BC was combined with other decreases in metastatic tumors associated with its inability to target the *TWIST1* EMT gene [119,167].

In addition, several proteins associated with BC invasiveness are capable of regulating some of the abovementioned miRNAs; here, TWIST1 acted as an inducer or repressor of the miR-10b and miR-200 cluster, SNAIL2 stimulated miR-221 up-regulation, and the proteolytically active ADAM8 activated miR-720 expression [81,119,158,165].

## 8. MicroRNAs and Exosome-Mediated Communication in Invasive Processes

While the activities of miRNAs expressed in breast cancer cells and stromal cells were presented in previous sections, miRNAs can also influence the invasive processes through their exosome-mediated transfer to other cells and be fully functional there [209]. 

Exosomes are defined as nanovesicles of endocytic origin and they are actively secreted from all types of cells. They transfer proteins, mRNAs and miRNAs and thereby facilitate many biological activities including cell-cell communication and cancer cell invasion. Moreover, current results indicate that tumor cell-derived exosomes have an important function in cancer progression, invasiveness and metastasis [210].

The levels of certain miRNAs in exosomes derived from human BC cell lines, such as miR-21 and miR-1246, were significantly increased, and these higher levels were found in plasma exosomes of BC patients compared to the plasma exosomes of healthy controls [211]. Serum level analysis of selected exosomal miRNAs in BC subtypes also returned higher concentrations of miR-373 in more aggressive TNBC [212].

The stimulation of invasive features in non-malignant breast cells through exosomes secreted by metastatic BC cells delivering miR-10b has been observed in cell line models. Function of miR-10b in non-malignant cells was documented by suppression of its target genes *HOXD10* and *KLF4* [213]. Furthermore, the recent study has shown that increased miR-21, miR-378e and miR-143 levels in exosomes obtained from CAFs compared to normal fibroblasts can contribute to the development of aggressive BC cell phenotype [214]. Other authors reported that miR-9, which is highly-expressed in many BC cell lines, influences the switch of normal fibroblasts to CAFs, and miR-9 secreted by tumor cells can be transported through exosomes to the recipient normal fibroblasts and result in increased cell motility. The up-regulation of miR-9 was also identified in CAFs isolated from TNBC patients compared to normal fibroblasts [215]. The participation of up-regulated miR-9, miR-10b, miR-21, and miR-373 in specific processes of BC invasiveness has previously been described in the miRNAs mentioned in this section.

Additionally, tumor-associated macrophages promote BC invasiveness through macrophages-secreted exosomes which can transport oncogenic miRNAs into BC cells [216]. Moreover, the-independent ability to process pre-miRNAs into mature miRNAs has also been identified in BC derived exosomes cells. The exosomes contain pre-miRNAs, with DICER, AGO2, and TRBP proteins, and those isolated from cells and sera of BC patients stimulate the normal epithelial cells to tumor development in a DICER-dependent manner [217].

## 9. Clinical Potential of microRNAs

Numerous investigations into the association of miRNAs and cancer has been performed over the last two decades, and important discoveries were made for miRNA biogenesis and cancer-specific alterations in miRNA expression in tumor samples. The miRNAs in circulation were identified as potential biomarkers, and there were also been some advances in miRNA-based cancer therapy [218]. Many researchers have attempted to utilize the aberrant miRNA expression profiles to identify disease states of cancer or subtypes, and the profiling of miRNAs has enhanced precise classification of many types of cancer [45,219]. For example, a set of miRNAs was identified in the early-stage of BC and different combinations of miRNAs were defined in two independent studies for the discrimination of the ER, progesterone receptor (PR), or HER2 status [219,220]. Many authors also performed miRNA quantifications using different detection platforms, and they made a great effort to utilise the gradually identified aberrant expression profiles of miRsupps and oncomiRs to develop new diagnostic, prognostic, or predictive biomarkers, as excellently reviewed in [42,221,222]. 

Several studies have shown dynamic changes during breast tumorigenesis and the dual roles of oncogenes and tumor suppressors of several miRNAs that could influence the evaluation of potential miRNA-based markers [48,49,50]. In addition, current studies focused on more detailed examination of individual miRNA target genes and the interactions between miRNAs and key oncogenes, tumor suppressors and regulation genes. Great research interest is also apparent in investigating the role of cancer-associated miRNA isoforms and exosome-mediated miRNA transfer to other cells in relationship to cancer invasiveness. These results all contribute to a more complex network which should be useful for new strategies in personalized medicine.

Significant progress has also been made in RNA-dependent therapy. Several tens of small interfering miRNAs with tumor suppressive effects and oncogenic antisense oligonucleotides have been tested to develop new anticancer drugs, and several miRNAs have currently been evaluated in phase I or II clinical trials. Mimics of miR-16, miR-29, and miR-34 have been utilized to target mesothelioma and non-small cell lung cancer, scleroderma, and multiple solid tumors, and the antimiRs 103/107, 122, and 155 have been tested and developed for treatment of diabetes, hepatitis C and cutaneous T-cell lymphoma, respectively [223]. However, only 16% of all developed anti-cancer therapeutic agents reach phase III trials, and the FDA usually approves only 10.4% of these [224]. Unfortunately, one-quarter of RNA-targeted therapeutics have not been investigated in human clinical trials and none of these pharmaceuticals have currently been approved by the FDA [225].

## 10. Conclusions

The growing list of identified human miRNAs raises great expectations that advanced investigations will improved understanding of the complexity of the miRNA regulation of gene expression in both normal and pathological processes. It is readily accepted that changes in miRNA expression profiles contribute to the development of many human diseases, including cancer. The high incidence of female malignancy-associated death, predominantly associated with BC, and the relatively high percentage of metastases highlight the urgent need for knowledge on a deeper molecular level for the following mechanisms: cancer cell detachment from the primary site, dissemination and propagation in secondary organs, and the miRNA regulation of the relevant genes. This will provide the basis for identification of precise diagnostic and prognostic markers and the development of new therapeutic molecules for the prevention and management of metastatic disease.

Our review summarised the most recent data on the expression of miRNAs and their targeted genes which function in processes involved in breast cancer invasiveness and cancer cell stemness. We then focused on the epigenetic deregulation of individual miRNAs and their modified interactions with other regulatory genes. In addition to many in silico, in vitro and in vivo studies, most of our evaluated miRNAs were also investigated in BC patient samples. The positive progress in metastasis research is reflected in the increasing number of cell-free miRNA expression studies of plasma samples from cancer patients which were not included in our review. The additional development of adequate assays for miRNA quantification in circulating tumor cells is expected to provide greater ability to identify exact markers of metastatic behaviour and improve the monitoring of cancer recurrence and therapy efficiency.

Finally, despite the variable detection methods for the quantification of miRNA expression and challenges imposed by analytic interpretation, our established results can successfully contribute to patient management and ultimately lead to prolongation of patients’ lives.

## Figures and Tables

**Table 1:** MicroRNAs participated in invasive processes and cancer cell stemness in breast cancer cells and patient’s tumor samples.

miRNA Name	Epigenetic Regulation	miRNA Expression in Tumor	Target Genes	Evaluated Samples	miRNA Role in Invasion-Metastasis Cascade	References
let-7		DOWN	*H-RAS, HMGA2*	CL, N = 25	Self-renewal, stemness	[178]
miR-7		DOWN	*PAK1*	CL, AM	Cell motility and invasiveness inhibition	[112]
		DOWN	*FAK*	CL, N = 111	Invasion and metastasis inhibition	[113]
miR-9		UP	*CDH1*	CL, AM, N = 23	Cell motility, invasion, metastasis	[102]
miR-10b		UP	*HOXD10*	CL, AM, N = 23	Cell migration and invasion	[81]
miR-21		UP	*TPM1*	CL	Tumor growth and malignant phenotype	[84]
		UP	*TIMP3*	CL, N = 32	Invasion and lymph node metastasis	[136]
miR-22		UP	*TET1-3*	CL, AM, N = 108	EMT, stemness	[186]
miR-23a		UP	*CDH1*	CL, AM, N = 30	EMT, metastasis	[148]
miR-29b		DOWN	*VEGFA, ANGPTL4, PDGF, LOX, MMP9*	CL, AM	EMC re-modeling and metastasis inhibition	[140]
miR-30a		DOWN	*ZEB2*	CL, N = 49	EMT, invasion and distal spreading inhibition (TNBC)	[157]
miR-30		DOWN	*UBC9*	CL, AM, N = 6	Self-renewal	[189]
miR-31		DOWN	*WAVE3*	CL, N = 19	Invasive phenotype reduction	[90]
		DOWN	*ITGA2, ITGA5, ITGAV. ITGB3*	CL	Invasion and metastasis inhibition	[108]
	DNA met	DOWN		CL	Invasion inhibition (TNBC)	[204]
miR-33a		DOWN	*ADAM9*	CL, AM, N = 23	Invasion and metastasis inhibition	[116]
miR-34		DOWN	*SNAI1*	CL	EMT inhibition	[159]
miR-34c	DNA met	DOWN	*NOTCH4*	CL	EMT, migration and self-renewal inhibition	[203]
miR-103/107		UP	*DICER1*	CL, AM	EMT, cell migration, metastasis	[73]
miR-124		DOWN	*SNAI2*	CL, AM, N = 38	EMT and metastasis inhibition	[160]
miR-124a	DNA met	DOWN		N = 60	Proliferation and metastasis inhibition	[206]
miR-126		DOWN	*ADAM9*	CL, N = 40	Invasion inhibition	[117]
miR-126/126*	DNA met	DOWN	*CXCL12*	CL, AM, N = 251	Metastasis inhibition	[127]
miR-140		DOWN	*SOX2*	CL, N = 8	Stemness, self-renewal	[194]
miR-142-3p		UP	*ITGAV, WASL, RAC1, CFL2*	CL	Cancer cell invasiveness inhibition	[110]
miR-145		DOWN	*JAM-A*	CL, N = 100	Cancer cell motility inhibition	[93,94]
miR-153		DOWN	*MTDH*	CL, N = 86	EMT and invasion inhibition	[170]
miR-154		DOWN	*ADAM9*	CL, N = 45	Cell migration and invasion inhibition	[118]
miR-155		UP	*RHOA*	CL, N = 62	EMT, tight junction dissolution, migration and invasion	[80]
miR-181a-5p		DOWN	*MMP14*	CL, N = 40	Cell migration inhibition	[135]
miR-181a		DOWN	*uPA*	CL	Invasion inhibition	[139]
miR-183 cluster		DOWN	*BMI1*	CL, AM, N = 11	Stemness, self-renewal	[176,177]
miR-191/425		UP	*DICER1*	CL, AM	Invasion and metastasis	[74]
miR-193b		DOWN	*uPA*	CL, AM, N = 80	Invasion and metastasis inhibition	[138]
miR-193a/b		DOWN	*uPA*	CL	Invasion inhibition	[139]
miR-198		DOWN	*CDCP1*	CL, N = 49	Cell adhesion and migration inhibition	[106]
miR-200 cluster (miR-200b/200a/429 and miR-200c/141)		DOWN	*WAVE3*	CL	Invasion and cell migration inhibition	[89]
		DOWN	*FLI1*, *TCF12*	CL, AM, N = 75	Cancer-associated fibroblast activation and ECM re-modeling inhibition	[131]
	DNA met	DOWN	*SNAI1, SNAI2, ZEB1, ZEB2*	CL, N = 70	EMT inhibition	[152]
		DOWN	*BMI1*	CL, AM, N = 11	Stemness, self-renewal	[176]
		DOWN	*SUZ12*	CL, AM, N = 8	Pluripotency	[181]
miR-200a		DOWN	*CX43*	CL, N = 40	Cell migration and metastasis inhibition	[87]
miR-200b		DOWN	*FERMT2*	CL, AM	EMT and metastasis inhibition	[154]
miR-200c		DOWN	*ZEB1, TKS5, MYLK*	CL, N = 101	EMT and invadopodia forming inhibition	[156]
miR-203		DOWN	*SNAI1*	CL	EMT, invasion and metastasis inhibition	[161]
	DNA met	DOWN	*SNAI2*	CL, N = 36	Invasion and migration inhibition	[200]
	DNA met	DOWN		CL, AM, N = 672	EMT and stem cells properties inhibition	[201]
miR-205		DOWN	*ZEB1, ZEB2*	CL	EMT inhibition	[163]
	DNA met	DOWN		CL	Invasion inhibition	[202]
miR-206		DOWN	*CX43*	CL, N = 60	Cell migration and metastasis inhibition	[88]
miR-221/222		DOWN	*ITGB4, STAT5A, ADAM17*	CL, N = 15	Invasion inhibition (luminal BC)	[114]
miR-221		UP	*CDH1*	CL, AM, N = 8	Invasion and metastasis	[165]
miR-221/222		UP	*TRPS1*	CL, N = 27	EMT, invasion and migration (basal-like BC)	[166]
miR-221		UP	*DNMT3B*	CL, N = 5	Pluripotency and stemness	[180]
miR-224		DOWN	*CDC42, CXCR4*	CL	Invasion, metastasis inhibition	[82]
DLK1-DIO3 cluster (miR-300/382/494/495/539/543/544)	DNA met	DOWN	*TWIST1, ZEB1, BMI1*	CL, N = 12	EMT and metastasis inhibition	[158]
miR-320		DOWN	*ETS2*	CL, AM, N = 126	Inhibition of tumor micro-environment re-programming	[130]
miR-335		DOWN	*TNC, SOX4*	CL, AM, N = 20	Invasion and metastasis inhibition	[133]
	DNA met	DOWN		CL, AM, N = 353	Metastasis inhibition	[205]
miR-373		UP	*ITGA2*	CL, N = 53	Cell migration, metastasis	[109]
miR-374a		UP	*WIF1, PTEN, WNT5A*	CL, AM, N = 166	EMT, metastasis	[149]
miR-375		DOWN	*SHOX2*	CL, N^#^	EMT inhibition	[171]
miR-494		DOWN	*PAK1*	CL, AM, N = 24	Clonogenic ability and cell migration, invasion and metastasis inhibition	[83]
		DOWN	*CXCR4*	CL	Cancer progression inhibition	[129]
miR-495		UP	*JAM-A*	CL, N = 7	Cancer cell migration	[95]
miR-506		DOWN	*SNAI2, VIM, CD151*	CL	EMT, adhesion, invasion, and migration inhibition	[169]
miR-539		DOWN	*LAMA4*	CL, N = 40	Cell migration inhibition (TNBC)	[132]
miR-590		DOWN	*SOX2*	CL, AM, N = 49	Stemness	[193]
miR-661		UP	*NECTIN1, STARD10*	CL, N = 295	EMT, invasion	[85]
miR-708		DOWN	*LSD1*	CL	Proliferation, invasion inhibition	[207]
	Histone met	DOWN	*NNAT*	CL, AM, N = 55	Migration and metastasis inhibition	[208]
miR-720		DOWN	*TWIST1*	CL, AM, N = 105	Invasion and metastasis inhibition	[167]

Abbreviations: met, methylation; CL, cell lines; AM, animal model; N, number of BC patients; BC, breast cancer; TNBC, triple negative breast cancer; ECM, extracellular matrix; EMT, epithelial-to-mesenchymal transition; N^#^, patient’s data from Gene Expression Omnibus. Notes: miR-126*, the partner to miR-126 that is derived from the same transcript, has a similar expression pattern [127].
